# Epigenome-wide association studies identify novel DNA methylation sites associated with PTSD: a meta-analysis of 23 military and civilian cohorts

**DOI:** 10.1186/s13073-024-01417-1

**Published:** 2024-12-18

**Authors:** Seyma Katrinli, Agaz H. Wani, Adam X. Maihofer, Andrew Ratanatharathorn, Nikolaos P. Daskalakis, Janitza Montalvo-Ortiz, Diana L. Núñez-Ríos, Anthony S. Zannas, Xiang Zhao, Allison E. Aiello, Allison E. Ashley-Koch, Diana Avetyan, Dewleen G. Baker, Jean C. Beckham, Marco P. Boks, Leslie A. Brick, Evelyn Bromet, Frances A. Champagne, Chia-Yen Chen, Shareefa Dalvie, Michelle F. Dennis, Segun Fatumo, Catherine Fortier, Sandro Galea, Melanie E. Garrett, Elbert Geuze, Gerald Grant, Michael A. Hauser, Jasmeet P. Hayes, Sian M. J. Hemmings, Bertrand Russel Huber, Aarti Jajoo, Stefan Jansen, Ronald C. Kessler, Nathan A. Kimbrel, Anthony P. King, Joel E. Kleinman, Nastassja Koen, Karestan C. Koenen, Pei-Fen Kuan, Israel Liberzon, Sarah D. Linnstaedt, Adriana Lori, Benjamin J. Luft, Jurjen J. Luykx, Christine E. Marx, Samuel A. McLean, Divya Mehta, William Milberg, Mark W. Miller, Mary S. Mufford, Clarisse Musanabaganwa, Jean Mutabaruka, Leon Mutesa, Charles B. Nemeroff, Nicole R. Nugent, Holly K. Orcutt, Xue-Jun Qin, Sheila A. M. Rauch, Kerry J. Ressler, Victoria B. Risbrough, Eugène Rutembesa, Bart P. F. Rutten, Soraya Seedat, Dan J. Stein, Murray B. Stein, Sylvanus Toikumo, Robert J. Ursano, Annette Uwineza, Mieke H. Verfaellie, Eric Vermetten, Christiaan H. Vinkers, Erin B. Ware, Derek E. Wildman, Erika J. Wolf, Ross McD Young, Ying Zhao, Leigh L. van den Heuvel, PGC-PTSD Epigenetics Workgroup, Seyma Katrinli, Jean C. Beckham, Segun Fatumo, Sandro Galea, Robert J. Ursano, Eric Vermetten, Reid S. Alisch, Ananda B Amstadter, Don Armstrong, Archana Basu, Nicole L Bjorklund, Barbara H Chaiyachati, Judith B M Ensink, Leland L Fleming, Joel Gelernter, Ryan J Herringa, Sonia Jain, Diana L Juvinao-Quintero, Elizabeth Ketema, José J Martínez-Magaña, Burook Misganaw, Shiela Tiemi Nagamatsu, Danny M Nispeling, John Pfeiffer, Christian Schmahl, Gen Shinozaki, Clara Snijders, Jennifer A Sumner, Patricia C Swart, Audrey Tyrka, Mirjam van Zuiden, Jaqueline S Womersley, Nagy A Youssef, Yuanchao Zheng, Yiwen Zhu, Lea Zillich, PsychENCODE PTSD Brainomics Project, Nikolaos P. Daskalakis, Frances A. Champagne, Aarti Jajoo, Joel E. Kleinman, Charles B. Nemeroff, Dhivya Arasappan, Sabina Berretta, Rahul A. Bharadwaj, Leonardo Collado-Torres, Christos Chatzinakos, Chris P. DiPietro, Duc M. Duong, Amy Deep-Soboslay, Nick Eagles, Louise Huuki, Thomas Hyde, Artemis Iatrou, Geo Pertea, Deanna Ross, Nicholas T. Seyfried, Joo Heon Shin, Traumatic Stress Brain Research Group, Bertrand Russel Huber, Victor E. Alvarez, David Benedek, Alicia Che, Dianne A. Cruz, David A. Davis, Matthew J. Girgenti, Ellen Hoffman, Paul E. Holtzheimer, Alfred Kaye, John H. Krystal, Adam T. Labadorf, Terence M. Keane, Ann McKee, Brian Marx, Crystal Noller, Meghan Pierce, William K. Scott, Paula Schnurr, Krista DiSano, Thor Stein, Douglas E. Williamson, Keith A. Young, Monica Uddin, Caroline M. Nievergelt, Alicia K. Smith, Mark W. Logue

**Affiliations:** 1 Department of Gynecology and Obstetrics, Emory University, Atlanta, GA USA; 2 Genomics Program, College of Public Health, University of South Florida, Tampa, FL USA; 3 Department of Psychiatry, University of California San Diego, La Jolla, CA San Diego, USA; 4 Center of Excellence for Stress and Mental Health, Veterans Affairs San Diego Healthcare System, San Diego, CA USA; 5 Research Service, Veterans Affairs San Diego Healthcare System, San Diego, CA USA; 6 Department of Epidemiology, Columbia University Mailmain School of Public Health, New York, NY USA; 7 Department of Epidemiology, Harvard T.H. Chan School of Public Health, Boston, MA USA; 8 Stanley Center for Psychiatric Research, Broad Institute of MIT and Harvard, Cambridge, MA USA; 9 Department of Psychiatry, Harvard Medical School, Boston, MA USA; 10 Center of Excellence in Depression and Anxiety Disorders, McLean Hospital, Belmont, MA USA; 11 U.S. Department of Veterans Affairs National Center of Posttraumatic Stress Disorder, Clinical Neurosciences Division, West Haven, CT USA; 12 VA Connecticut Healthcare System, West Haven, CT USA; 13 Department of Psychiatry, Yale School of Medicine, New Haven, CT USA; 14 Carolina Stress Initiative, University of North Carolina at Chapel Hill, NC Chapel Hill, USA; 15 Department of Genetics, University of North Carolina at Chapel Hill, NC Chapel Hill, USA; 16 Department of Psychiatry, University of North Carolina at Chapel Hill, NC Chapel Hill, USA; 17 Institute for Trauma Recovery, University of North Carolina at Chapel Hill, NC Chapel Hill, USA; 18 Department of Biostatistics, Boston University School of Public Health, Boston, MA USA; 19 Robert N. Butler Columbia Aging Center, Department of Epidemiology, Columbia University, New York, NY USA; 20 Duke Molecular Physiology Institute, Duke University Medical Center, Durham, NC USA; 21 Psychiatry Service, Veterans Affairs San Diego Healthcare System, San Diego, CA USA; 22 Department of Psychiatry and Behavioral Sciences, Duke University School of Medicine, Durham, NC USA; 23 Durham VA Health Care System, Researcg, Durham, NC USA; 24 Genetics Research Laboratory, VA Mid-Atlantic Mental Illness Research Education, and Clinical Center (MIRECC), Durham, NC USA; 25 Department of Psychiatry, Brain Center University Medical Center Utrecht, Utrecht, UT, NL Netherlands; 26 Department of Psychiatry and Human Behavior, Alpert Medical School of Brown University, Providence, RI USA; 27 Epidemiology Research Group, State University of New York at Stony Brook, Stony Brook, NY USA; 28 Department of Psychology, The University of Texas at Austin, Austin, TX USA; 29 Biogen Inc, Translational Sciences, Cambridge, MA USA; 30 Department of Pathology, University of Cape Town, Western Province, Cape Town, ZA South Africa; 31 Division of Human Genetics, University of Cape Town, Western Province, Cape Town, ZA South Africa; 32 MRC/UVRI and London School of Hygiene and Tropical Medicine, The African Computational Genomics (TACG) Research Group, Entebbe, Wakiso, Uganda; 33 Translational Research Center for TBI and Stress Disorders (TRACTS)/Geriatric Research Education and Clinical Center (GRECC), VA Boston Healthcare System, Boston, MA USA; 34 School of Public Health, Boston University, Boston, MA USA; 35 Brain Research and Innovation Centre, Netherlands Ministry of Defence, Utrecht, UT, NL Netherlands; 36 Department of Psychiatry, UMC Utrecht Brain Center Rudolf Magnus, Utrecht, UT Netherlands; 37 Department of Neurosurgery, Duke University School of Medicine, Durham, NC USA; 38 Department of Medicine, Duke University School of Medicine, Durham, NC USA; 39 Department of Psychology, The Ohio State University, Columbus, OH USA; 40 Department of Psychiatry, Faculty of Medicine and Health Sciences, Stellenbosch University, Western Cape, Cape Town, ZA South Africa; 41 SAMRC Genomics of Brain Disorders Research Unit, Stellenbosch University, Western Cape, Cape Town, ZA South Africa; 42 Department of Neurology, Boston University School of Medicine, Boston, MA USA; 43 Pathology and Laboratory Medicine, VA Boston Healthcare System, Boston, MA USA; 44 McLean Hospital, Belmont, MA USA; 45 College of Medicine and Health Sciences, University of Rwanda, Kigali, RW Rwanda; 46 Department of Health Care Policy, Harvard Medical School, Boston, MA USA; 47 Mental Health Service Line, Durham VA Health Care System, Durham, NC USA; 48 Institute for Behavioral Medicine Research, The Ohio State University College of Medicine, Columbus, OH USA; 49 Psychiatry & Behavioral Health, The Ohio State University College of Medicine, Columbus, OH USA; 50 Department of Psychiatry and Behavioral Sciences, Johns Hopkins University School of Medicine, Baltimore, MD USA; 51 Lieber Institute for Brain Development, Baltimore, MD USA; 52 Department of Psychiatry & Mental Health, University of Cape Town, Western Province, Cape Town, ZA South Africa; 53 Neuroscience Institute, University of Cape Town, Western Province, Cape Town, ZA South Africa; 54 SA MRC Unit on Risk & Resilience in Mental Disorders, University of Cape Town, Western Province, Cape Town, ZA South Africa; 55 Psychiatric and Neurodevelopmental Genetics Unit (PNGU), Massachusetts General Hospital, Boston, MA USA; 56 Department of Applied Mathematics and Statistics, Stony Brook University, Stony Brook, NY USA; 57 Department of Psychiatry and Behavioral Sciences, Texas A&M University College of Medicine, Bryan, TX USA; 58 Department of Anesthesiology, University of North Carolina at Chapel Hill, NC Chapel Hill, USA; 59 UNC Institute for Trauma Recovery, University of North Carolina at Chapel Hill, NC Chapel Hill, USA; 60 Department of Psychiatry and Behavioral Sciences, Emory University, Atlanta, GA USA; 61 Department of Medicine, Stony Brook University, Stony Brook, NY USA; 62 Amsterdam Neuroscience Research Institute Stress & Sleep Program, Amsterdam University Medical Center, Amsterdam, NH Netherlands; 63 Amsterdam Public Health Research Institute, Mental Health Program, Amsterdam University Medical Center, Amsterdam, NH Netherlands; 64 Department of Psychiatry, Amsterdam University Medical Center, Amsterdam, NH Netherlands; 65 Durham VA Health Care System, Durham, NC USA; 66 VA Mid-Atlantic Mental Illness Research Education, and Clinical Center (MIRECC), Durham, NC USA; 67 Department of Psychiatry, UNC Institute for Trauma Recovery, NC Chapel Hill, USA; 68 Centre for Genomics and Personalised Health, Queensland University of Technology, Kelvin Grove, QLD, AU Brisbane, Australia; 69 School of Biomedical Sciences, Queensland University of Technology, Kelvin Grove, QLD, AU Brisbane, Australia; 70 GRECC/TRACTS, VA Boston Healthcare System, Boston, MA USA; 71 Biomedical Genetics & Psychiatry, Boston University Chobanian & Avedisian School of Medicine, Boston, MA USA; 72 National Center for PTSD, VA Boston Healthcare System, Boston, MA USA; 73 Department of Psychiatry and Mental Health, University of Cape Town, Western Province, Cape Town, ZA South Africa; 74 Research Innovation and Data Science Division, Rwanda Biomedical Center, Kigali, Rwanda; 75 Center of Human Genetics, University of Rwanda, Kigali, RW Rwanda; 76 Department of Clinical Psychology, University of Rwanda, Huye, RW Rwanda; 77 Center for Human Genetics, University of Rwanda, Kigali, RW Rwanda; 78 Department of Psychiatry and Behavioral Sciences, The University of Texas at Austin, Austin, TX USA; 79 Department of Emergency Medicine, Alpert Brown Medical School, Providence, RI USA; 80 Department of Pediatrics, Alpert Brown Medical School, Providence, RI USA; 81 Department of Psychiatry and Human Behavior, Alpert Brown Medical School, Providence, RI USA; 82 Department of Psychology, Northern Illinois University, DeKalb, IL USA; 83 Duke Molecular Physiology Institute, Duke University, Durham, NC USA; 84 Department of Psychiatry & Behavioral Sciences, Emory University, Atlanta, GA USA; 85 Joseph Maxwell Cleland Atlanta Veterans Affairs Healthcare System, Atlanta, GA USA; 86 Clinical Psychology, University of Rwanda, Kigali, RW Rwanda; 87 Department of Psychiatry and Neuropsychology, School for Mental Health and Neuroscience, Maastricht Universitair Medisch Centrum, Maastricht, Limburg, NL Netherlands; 88 SA MRC Extramural Genomics of Brain Disorders Research Unit, Stellenbosch University, Western Cape, Cape Town, ZA South Africa; 89 School of Public Health, University of California San Diego, CA La Jolla, USA; 90 SA MRC Genomics of Brain Disorders Research Unit, Stellenbosch University, Western Cape, Cape Town, ZA South Africa; 91 Department of Psychiatry, Center for the Study of Traumatic Stress, Uniformed Services University, Bethesda, MD USA; 92 College of Medicine and Health Sciences, University of Rwanda, Kigali, Rwanda; 93 Department of Psychiatry, Boston University School of Medicine, Boston, MA USA; 94 Memory Disorders Research Center, VA Boston Healthcare System, Boston, MA USA; 95 Department of Psychiatry, Leiden University Medical Center, Leiden, ZH, NL Netherlands; 96 Department of Psychiatry, New York University School of Medicine, New York, NY USA; 97 Department of Anatomy and Neurosciences, Amsterdam UMC Location Vrije Universiteit Amsterdam, Amsterdam, Holland, Netherlands; 98 Department of Psychiatry, Amsterdam, UMC Location Vrije Universiteit Amsterdam, Amsterdam, Holland, Netherlands; 99 Amsterdam University Medical Center, Amsterdam Neuroscience Research Institute, Stress & Sleep Program, MoodPsychosisAmsterdam, Holland, AnxietyNL Netherlands; 100 Survey Research Center, University of Michigan, Ann Arbor, MI USA; 101 College of Public Health, University of South Florida, Tampa, FL USA; 102 Genomics Program, University of South Florida, Tampa, FL USA; 103 Department of Psychiatry, Boston University Chobanian & Avedisian School of Medicine, Boston, MA USA; 104 School of Clinical Sciences, Queensland University of Technology, Kelvin Grove, QLD, AU Brisbane, Australia; 105 University of the Sunshine Coast, The Chancellory Sippy Downs, QLD, AU Buderim, Australia; 106 University of South Florida College of Public Health, Genomics Program, Tampa, FL USA; 107 Department of Human Genetics, Emory University, Atlanta, GA USA

**Keywords:** PTSD, Trauma, DNA methylation, Postmortem brain, GWAS, Gene expression

## Abstract

**Background:**

The occurrence of post-traumatic stress disorder (PTSD) following a traumatic event is associated with biological differences that can represent the susceptibility to PTSD, the impact of trauma, or the sequelae of PTSD itself. These effects include differences in DNA methylation (DNAm), an important form of epigenetic gene regulation, at multiple CpG loci across the genome. Moreover, these effects can be shared or specific to both central and peripheral tissues. Here, we aim to identify blood DNAm differences associated with PTSD and characterize the underlying biological mechanisms by examining the extent to which they mirror associations across multiple brain regions.

**Methods:**

As the Psychiatric Genomics Consortium (PGC) PTSD Epigenetics Workgroup, we conducted the largest cross-sectional meta-analysis of epigenome-wide association studies (EWASs) of PTSD to date, involving 5077 participants (2156 PTSD cases and 2921 trauma-exposed controls) from 23 civilian and military studies. PTSD diagnosis assessments were harmonized following the standardized guidelines established by the PGC-PTSD Workgroup. DNAm was assayed from blood using Illumina HumanMethylation450 or MethylationEPIC (850 K) BeadChips. Within each cohort, DNA methylation was regressed on PTSD, sex (if applicable), age, blood cell proportions, and ancestry. An inverse variance-weighted meta-analysis was performed. We conducted replication analyses in tissue from multiple brain regions, neuronal nuclei, and a cellular model of prolonged stress.

**Results:**

We identified 11 CpG sites associated with PTSD in the overall meta-analysis (1.44e − 09 < *p* < 5.30e − 08), as well as 14 associated in analyses of specific strata (military vs civilian cohort, sex, and ancestry), including CpGs in *AHRR* and *CDC42BPB*. Many of these loci exhibit blood–brain correlation in methylation levels and cross-tissue associations with PTSD in multiple brain regions. Out of 9 CpGs annotated to a gene expressed in blood, methylation levels at 5 CpGs showed significant correlations with the expression levels of their respective annotated genes.

**Conclusions:**

This study identifies 11 PTSD-associated CpGs and leverages data from postmortem brain samples, GWAS, and genome-wide expression data to interpret the biology underlying these associations and prioritize genes whose regulation differs in those with PTSD.

**Supplementary Information:**

The online version contains supplementary material available at 10.1186/s13073-024-01417-1.

## Background

Posttraumatic stress disorder (PTSD) is a serious psychiatric disorder characterized by intrusive memories of the traumatic event(s), avoidance of or numbing to situations that trigger those memories, and hyperarousal symptoms that can disturb mental and physical health [1]. These symptoms are associated with lower levels of self-care, lower compliance with medical treatment, and higher rates of substance use [2, 3]. Thus, it is not surprising that PTSD increases the risk for chronic medical conditions, such as cardiovascular disorders, independent of lifestyle factors (e.g., substance use and sleep quality) [4, 5]. Although most individuals experience at least one traumatic event, only a small fraction develop PTSD [6]. Genetic and environmental factors contribute to this differential susceptibility in PTSD development upon trauma exposure [7, 8].

Genome-wide association studies (GWAS) of PTSD demonstrated remarkable success at identifying relevant genes, many of which are involved in the stress response or immune function (see reviews[9, 10]). The recent Psychiatric Genomics Consortium PTSD Workgroup (PGC-PTSD) Freeze 3 GWAS identified 95 genomic loci associated with PTSD, implicating genes involved in stress, immune, fear, and threat-related processes [11]. Nonetheless, genetic differences cannot fully account for an individual’s susceptibility to PTSD. Trauma exposure has been shown to alter epigenetic patterns in both animal and human studies, prompting the need to conduct epigenetic studies of PTSD in addition to genetic studies [12, 13]. Epigenetic mechanisms are chemical modifications that can dictate the timing and magnitude of gene expression without altering the DNA sequence [14]. The most widely studied epigenetic mechanism is DNA methylation (DNAm), which is defined as the addition of a methyl group to cytosine bases, particularly at cytosine-guanine dinucleotides (CpG sites). DNAm patterns respond to changes in the environment, are potentially reversible, and can be targeted for disease therapies [15, 16]. Environmental influences on DNAm are apparent across the lifespan and may provide insight into the biological response to trauma [17].

Which specific DNAm sites differ across individuals and how they correlate with exposures and gene expression can vary across tissues [18]. DNAm in human brain tissue, which is most relevant to the study of psychiatric disorders, is not easily accessible in living patients and hence is not a viable PTSD biomarker for clinical use. However, correlation has been observed between peripheral tissues (e.g., blood) and brain DNAm levels at specific genomic loci, and thus, blood DNAm can potentially serve as a robust biomarker for implementing early intervention and developing improved preventative or therapeutic strategies for PTSD [19, 20]. Moreover, PTSD symptoms have been linked to the components of the peripheral immune system [21, 22] that can be readily assessed in blood DNAm. Multiple peripheral epigenome-wide association studies (EWASs) of PTSD identified CpGs in genes related to the immune system and neurotransmission [23–28]. While prior EWASs of PTSD have reported promising results, the small sample sizes and variability of analysis methods across studies make it difficult to combine and interpret the findings effectively. Recent meta-analyses led by the PGC-PTSD Epigenetics Workgroup minimized these issues by increasing sample size, increasing sample diversity, and using a consortium-supplied quality control and analysis pipelines [29–33]. These meta-analyses identified multiple new loci associated with PTSD, including *NRG1*,* AHRR*,* MAD1L1*, and *TBXAS1*, implicating immune dysregulation in those with PTSD [30–33].

Building on the prior work by Smith et al. [31], which conducted an EWAS meta-analysis in 1896 participants from 10 cohorts, this study includes 13 additional cohorts with a denser and more comprehensive DNAm array, bringing the sample up to 5077 participants from 23 civilian and military cohorts. Our current investigation replicated the findings of the initial PGC-PTSD epigenome-wide meta-analysis, reporting lower *AHRR* methylation in those with PTSD, and identified 9 new (11 total) loci associated with PTSD, as well as 14 CpGs associated in analyses of specific strata (military vs civilian cohort, sex, and ancestry). We also leveraged data from postmortem brain samples, a cellular model of prolonged stress, GWAS, and genome-wide gene expression studies to interpret the biology underlying these associations and prioritize genes whose regulation differs in those with PTSD.

## Methods

### Cohorts and post-traumatic stress disorder assessments

The study includes 2156 current PTSD cases and 2921 trauma-exposed controls from 9 civilian cohorts: BEAR, DNHS, DCHS, GTP, NIU, Shared Roots, AURORA, H3A_Rwanda, WTC; and 9 military cohorts: GMRFQUT, MRS, PRISMO, Army STARRS, PROGrESS, NCPTSD/TRACTS, INTRuST, and VA cohorts (VA-M-AA and VA-M-EA). For DNHS, GTP, MRS, PRISMO, and Army STARRS, two different datasets were available based on the DNAm array. Two different datasets for these five cohorts did not have any overlapping samples and were treated as independent studies. Sample characteristics for the 23 studies that participated in the meta-analysis are summarized in Table 1. Detailed descriptions of each cohort were presented in Additional file 1: eMethods.

**Table 1:** Overview of the studies

Cohort	Array	*N*	Cases *N* (%)	Controls *N* (%)	Female *N* (%)	European *N* (%)	African *N* (%)	Age Mean (SD)
**Civilian**								
BEAR	EPIC	162	36 (22%)	126 (78%)	119 (73%)	112 (69%)	3 (2%)	15.16 (1.45)
DNHS-1	EPIC	423	26 (6%)	397 (94%)	255 (60%)	23 (5%)	384 (91%)	54.54 (16.87)
DCHS	EPIC	95	46 (48%)	49 (52%)	95 (100%)	0 (0%)	54 (57%)	26.81 (5.2)
GTP-1	EPIC	479	158 (33%)	321 (67%)	340 (71%)	12 (3%)	448 (94%)	42.22 (12.25)
NIU	EPIC	140	18 (13%)	122 (87%)	140 (100%)	110 (79%)	19 (14%)	26.01 (1.74)
Shared Roots	EPIC	120	61 (51%)	59 (49%)	85 (71%)	0 (0%)	120 (100%)	43.15 (10.77)
AURORA	EPIC	206	57 (28%)	149 (72%)	154 (75%)	67 (33%)	131 (64%)	39.24 (14.17)
H3A_Rwanda	EPIC	73	32 (44%)	41 (56%)	73 (100%)	0 (0%)	73 (100%)	45.54 (7.29)
DNHS-2	450 K	100	40 (40%)	60 (60%)	60 (60%)	13 (13%)	87 (87%)	53.6 (14.01)
GTP-2	450 K	265	74 (28%)	191 (72%)	187 (71%)	16 (6%)	249 (94%)	41.95 (12.37)
WTC	450 K	180	84 (47%)	96 (53%)	0 (0%)	138 (77%)	7 (4%)	49.72 (8.25)
* Civilian total*		*2243*	*632 (28%)*	*1611 (72%)*	*1508 (67%)*	*491 (22%)*	*1575 (70%)*	*41.89 (16.27)*
**Military**								
GMRFQUT	EPIC	96	48 (50%)	48 (50%)	0 (0%)	96 (100%)	0 (0%)	68.67 (4.36)
MRS-1	EPIC	127	64 (50%)	63 (50%)	0 (0%)	88 (69%)	5 (4%)	23.07 (2.18)
PRISMO-1	EPIC	89	24 (27%)	65 (73%)	9 (10%)	74 (83%)	3 (3%)	27.51 (8.63)
Army STARRS-1	EPIC	216	106 (49%)	110 (51%)	0 (0%)	149 (69%)	22 (10%)	25.13 (4.82)
PROGrESS	EPIC	140	112 (80%)	28 (20%)	14 (10%)	89 (64%)	40 (29%)	34.77 (8.33)
NCPTSD/TRACTS	EPIC	1028	638 (62%)	390 (38%)	231 (22%)	706 (69%)	123 (12%)	44.06 (13.7)
MRS-2	450 K	126	63 (50%)	63 (50%)	0 (0%)	72 (57%)	10 (8%)	22.2 (3.04)
PRISMO-2	450 K	62	32 (52%)	30 (48%)	0 (0%)	62 (100%)	0 (0%)	27.1 (9.23)
Army STARRS-2	450 K	102	51 (50%)	51 (50%)	0 (0%)	102 (100%)	0 (0%)	23.79 (4.25)
INTRuST	450 K	303	116 (38%)	187 (62%)	102 (34%)	206 (68%)	58 (19%)	34.09 (11.68)
VA-M-AA	450 K	369	183 (50%)	186 (50%)	184 (50%)	0 (0%)	369 (100%)	38.36 (9.36)
VA-M-EA	450 K	176	87 (49%)	89 (51%)	38 (22%)	176 (100%)	0 (0%)	34.87 (9.89)
* Military total*		*2834*	*1524 (54%)*	*1310 (46%)*	*578 (20%)*	*1820 (64%)*	*630 (22%)*	*37.08 (14.33)*
**Total**		**5077**	**2156 (42%)**	**2921 (58%)**	**2086 (41%)**	**2311 (46%)**	**2205 (43%)**	**39.2 (15.4)**

Participating civilian cohorts: biomarkers, social, and affective predictors of suicidal thoughts and behaviors in adolescents (BEAR), Detroit Neighborhood Health Study (DNHS), Drakenstein Child Health Study (DCHS), Grady Trauma Project (GTP), Northern Illinois University Trauma Study (NIU), Shared Roots, Advancing Understanding of RecOvery afteR traumA (AURORA), Human Heredity and Health in Africa, Rwanda (H3A_Rwanda), World Trade Center 9/11 Responders (WTC). Participating military cohorts: Gallipoli Medical Research Foundation Queensland University of Technology (GMRFQUT), Marine Resiliency Study (MRS), Prospective Research in Stress-related Military Operations (PRISMO), Army Study to Assess Risk and Resilience in Servicemembers (Army STARRS), PROlonGed ExpoSure and Sertraline Trial (PROGrESS), Boston VA—National Center for PTSD/ Translational Research Center for TBI and Stress Disorders (NCPTSD/TRACTS), Injury and Traumatic Stress study (INTRuST), and Veterans Affairs’ Mental Illness Research, Education and Clinical Centers (VA-M-AA and VA-M-EA). Note: For DNHS, GTP, MRS, PRISMO, and Army STARRS cohorts, EPIC and 450 k datasets represent different sets of participants

*Biomarkers, social, and affective predictors of suicidal thoughts and behaviors in adolescents (BEAR)* [34] involved a sample of 194 adolescents who had been hospitalized for suicidal thoughts/behaviors. Of those, 163 provided a blood sample, and 162 samples that passed the DNAm quality control (QC) were included in the meta-analysis. PTSD diagnosis was assessed by the Clinician Administered PTSD Scale (CAPS) for Children and Adolescents for DSM-5 [35].

*Detroit Neighborhood Health Study (DNHS)* [23] involved 1547 participants whose PTSD symptoms were assessed by the PTSD checklist (PCL-C) [36] at the baseline wave. The meta-analysis included 523 participants with available DNAm data that passed the DNAm QC.

*Drakenstein Child Health Study (DCHS)* [37] is a population-based birth cohort that recruited 1000 pregnant people between 20–28 weeks gestation. DNAm data was available for 98 participants, and 95 samples that passed the DNAm QC were included in the meta-analysis. For the purpose of this study, PTSD was assessed using The Mini International Neuropsychiatric Interview (MINI) [38, 39].

*Grady Trauma Project (GTP)* is a large-scale ongoing study with > 10,000 participants. The meta-analysis included 744 participants with available DNAm data that passed the DNAm QC. Current PTSD was assessed using the Clinician-Administered PTSD Scale for DSM IV (CAPS-4) [40, 41] or the modified PTSD Symptomatic Scale (PSS) [42].

*Northern Illinois University Trauma Study (NIU)* involved 812 participants recruited to participate in a federally-funded study (NIH 5R21MH085436-02) to examine risk and protective factors following the NIU campus shooting on February 14th, 2008. Of those, 140 provided blood samples and were included in the meta-analysis. Current PTSD diagnosis at the first post-shooting assessment was assessed by self-report on the Distressing Events Questionnaire (DEQ) [43].

*Shared Roots* SHRS or “Understanding the SHARED ROOTS of Neuropsychiatric Disorders and Modifiable Risk Factors for Cardiovascular Disease” is a matched case–control study (*N* = 120) examining the factors that contribute to the comorbidity of metabolic syndrome and neuropsychiatric disorders [44]. The CAPS-5 [45] was administered by clinicians to assess PTSD over the prior month.

*Advancing Understanding of RecOvery afteR traumA (AURORA)* is a large cohort study of women and men presenting to the ED within 72 h after exposure to psychological trauma [46]. PTSD diagnosis at 6 months was defined using the PTSD Checklist for DSM-5 (PCL-5) [47–49]. The meta-analysis included a subset of the AURORA cohort with available phenotypic, DNA methylation, and RNA sequencing data at the 6-month follow-up after trauma exposure (*N* = 206).

*Human Heredity and Health in Africa, Rwanda (H3A_Rwanda).* Data from the H3A_Rwanda cohort were obtained from a subset of participants in a previously published pilot study (*n* = 50) [50, 51] and newly recruited participants via support from the H3Africa Consortium (*n* = 40) [52]. All study participants were women of Tutsi ethnicity who were pregnant during the genocide. PTSD was assessed by PCL-17 [53] for the pilot study and by PCL-5 [54] for the H3Africa-affiliated study. The meta-analysis included 73 participants with available phenotype and DNAm data.

*World Trade Center Responders (WTC)* [55, 56]. PTSD in relation to WTC exposures was assessed by the Structured Clinical Interview for DSM-IV Disorders (SCID) [57]. The sample (*N* = 180) providing blood samples for the epigenetics assays was restricted to men (the vast majority of the responders) and oversampled for posttraumatic stress disorder (PTSD) [25].

*Gallipoli Medical Research Foundation Queensland University of Technology (GMRFQUT)* is a large cohort of veterans who have been or are currently being treated for PTSD at the Keith Payne Unit within the Greenslopes Private Hospital in Queensland, Australia [58]. The CAPS-5 [40, 41] was used to assess current PTSD. The meta-analysis included 96 participants with available DNAm data.

*Marine Resiliency Study (MRS).* In the MRS [59, 60], PTSD was diagnosed by CAPS-5 [40, 41] up to 3 times, once before deployment and 3 and/or 6 months post-deployment. Samples of PTSD cases (*N* = 127) were selected from the 3- or 6-month post-deployment visits depending on which visit had the highest CAPS score. Combat-exposed controls with low to no PTSD-symptoms (*N* = 126) were selected from post-deployment visits, matching for age, ancestry, and time of post-deployment visit.

*Prospective Research in Stress-related Military Operations (PRISMO)* is a large prospective study of Dutch military soldiers [61, 62]. Current PTSD symptoms were assessed using the Self-Report Inventory for PTSD (SRIP) [63], and blood samples were collected approximately 1 month before and at both 1 and 6 months after deployment. PTSD cases (*N* = 56) were selected for this DNA methylation study from the 1- or 6-month post-deployment visits, depending on which visit had the highest SRIP score. All controls (*N* = 95) were combat exposed and had low PTSD symptoms [26].

*The Army Study to Assess Risk and Resilience in Servicemembers (Army STARRS)* is a multicomponent prospective study among US Army personnel [64]. PTSD diagnosis was assigned using multiple imputation methods that relied on PCL and CIDI-SC data [65]. Whole blood for methylation assays was collected approximately 6 weeks before deployment and 1-month post-deployment. PTSD cases (*N* = 157) were selected based on their PTSD diagnosis at 6 months post-deployment. Controls (*N* = 161) were participants without PTSD, matched on age, deployment stress, and childhood adversity.

*PROlonGed ExpoSure and Sertraline Trial (PROGrESS)* is a randomized-controlled trial (RCT; *N* = 223) designed to examine the comparative effectiveness of multiple treatment strategies across 24 weeks. PTSD is assessed by the CAPS-IV [40, 41]. This study included 112 PTSD cases and 28 trauma-exposed controls selected from the pre-treatment visit.

*Boston VA National Center for PTSD/Translational Research Center for TBI and Stress Disorders (NCPTSD/TRACTS)* included participants from the NCPTSD study [66], the PTSD and Accelerated Aging study [67], and the TRACTS study [68]. For all three studies, PTSD diagnosis was determined based on the CAPS for DSM-IV [40] or DSM-5 [69]. The NCPTSD and TRACTS cohort DNAm (*N* = 1028) data were jointly cleaned and analyzed together.

*Injury and Traumatic Stress (INTRuST)*. This cohort included participants in studies of the INTRuST Consortium [70]. PTSD diagnosis was assessed by study-specific measures, including CAPS-IV, CAPS-5 [40, 41], and PCL-17 [53]. The meta-analysis included 303 participants with available phenotype and DNAm data.

*Mid-Atlantic Mental Illness Research Education and Clinical Center PTSD Study (VA-M-AA & VA-M-EA)* [71–73]. PTSD was diagnosed using the SCID [57]. The meta-analysis included 369 participants from VA-M-AA and 87 participants from VA-M-EA with available phenotype and DNAm data.

The sample is heterogeneous in terms of sex (41% female), ancestry (46% European, 43% African, and 11% of other ancestries), and cohort type (56% military cohort). Civilian cohorts skew towards being more female (67%) and African (70%), whereas military cohorts are predominantly male (80%) and European (64%). In addition, the prevalence of PTSD is higher in military cohorts (54%) than in civilian cohorts (28%).

All participants were exposed to a traumatic event and 42% met the criteria for current PTSD. The current PTSD diagnosis was assessed by each study following the standardized guidelines established by the PGC-PTSD Workgroup [7]. Briefly, current PTSD diagnosis was determined based on the specific criteria set by the principal investigator of each study. Participants without current PTSD but with a history of PTSD (i.e., remitted PTSD), were excluded. Detailed descriptions of cohorts and PTSD assessments are provided in the Additional file 1: eMethods. All participants in these studies gave informed consent. The institutional review boards of each respective institution approved these studies.

### DNA methylation

Whole blood DNAm was measured using the Illumina MethylationEPIC BeadChip (EPIC array) in 14 studies, and the HumanMethylation450 BeadChip (450 K array) in 9 studies (Table 1). All studies used a standardized consortium-developed QC pipeline that differed somewhat depending on which chip was used. The 450 K array pipeline [29, 31] is described in Additional file 1: eMethods.

The EPIC pipeline (available at https://github.com/PGC-PTSD-EWAS/EPIC_QC) [74] was similar to the 450 K array pipeline. Samples with probe detection call rates lower than 90% and average intensity values that were either less than 50% of the overall sample mean or below 2000 arbitrary units (AU) were excluded. Probes with detection *p*-values > 0.01 were considered low quality and treated as missing. Probes that were missing in > 10% of the samples within the studies and were cross-hybridizing were removed [75]. Data was normalized using single-sample Noob (ssNoob) implemented in R package *minfi* [76]. *ComBat* was used to account for batch effects of chip and position while preserving PTSD, age, and sex effects (if applicable) [77]. Blood-cell composition (i.e., the proportion of CD8 + T, CD4 + T, natural killer (NK), B cells, monocytes, and neutrophils) was estimated using the robust partial correlation (RPC) method in *Epidish* [78] with a reference data specific to EPIC array [79]. For studies without genome-wide genotype data (DNHS, NIU, Shared Roots, AURORA, H3A_Rwanda, GMRFQUT, PROGrESS), we estimated ancestry principal components (PCs) from DNAm data, using the method developed by Barfield et al. [80], as previously described [33]. PCs 2 and 3, which were the components that correlate most with self-reported ancestry, were included as covariates [33, 80]. In cohorts with available genome-wide genotype data, PCs 1–3 from GWAS were used to adjust for ancestry. We used R package *bacon* to control inflation, only if doing so results in the lambda being closer to 1 [81]. To predict smoking status, a DNAm-based smoking score was calculated, as previously described [27] for cohorts with EPIC array data. A detailed description of DNAm-based ancestry PC and smoking score calculation is provided in Additional file 1: eMethods.

### Epigenome-wide association analysis

The association between PTSD and DNAm was tested using multivariable linear regression models for cohorts with balanced plate designs. For studies in which plate layouts were not balanced (Shared Roots, H3A_Rwanda, GMRFQUT), we conducted mixed-effect regression models, including chip as a random effect term. The *CpGassoc* R package was used to fit the models [82]. The models were adjusted for age, sex (if applicable), blood cell composition (i.e., CD8T, CD4T, NK, B cell, and monocyte cell proportions), and ancestry PCs. While not included initially because of concerns about multicoliniarity and collider bias, a post hoc sensitivity analysis was performed including a covariate for smoking: DNAm-based smoking score in studies with EPIC data and current smoking status for studies with 450 K data. Furthermore, we conducted stratified analyses for both sexes, ancestry (European and African ancestry), and cohort type (civilian and military cohorts).

To combine results across studies, we performed inverse-variance weighted (IVW) meta-analysis in *meta* [83]. Meta-analysis tested 411,786 CpGs common to 450 K and EPIC arrays (23 studies), and 404,794 EPIC array specific CpGs (14 studies). Epigenome-wide significance threshold recommended for the MethylationEPIC BeadChip (*p* < 9.0e − 08) was used to determine statistical significance [84]. Gene Ontology (GO) enrichment analyses were conducted using the top 1000 CpGs in *missMethyl* [85]. A false discovery rate (FDR) threshold of 5% was used to identify significant GO terms.

### Gene regulation

Correlations between PTSD-associated CpG sites’ DNAm levels and expression levels of the corresponding gene (as determined by the EPIC v1 array annotation) were tested in whole-blood RNA-sequencing (RNA-seq) data from participants in the BEAR (*n* = 127), AURORA (*n* = 173), NCPTSD Merit (*n* = 204), and MRS cohorts (*n* = 128 with multiple visits totaling 357 samples). The results were meta-analyzed using the IVW method using a Bonferroni correction for the CpGs examined. Detailed information about cohort-specific RNA-seq data generation is described in Additional file 1: eMethods.

### Genetic effects

To evaluate the effect of nearby (< 1 MB) polymorphisms on DNAm levels of CpGs associated with PTSD, we used cis-methylation quantitative trait locus (cis-meQTL) data from GoDMC [86] and meQTL EPIC [87] databases. For both databases, their default multiple testing adjustment was utilized: an FDR threshold of 5% in meQTL EPIC and *p* < 1e − 08 in GoDMC. We checked the associations between the identified cis-SNPs and PTSD in the recent Freeze 3 GWAS from PGC-PTSD [11]. Finally, we evaluated genetic associations between the genes with PTSD-associated DNAm and PTSD, using the gene-based test results from the recent PGC-PTSD Freeze 3 GWAS [11].

### Cross-tissue association analyses

#### Blood–brain correlations

The Blood Brain DNA Methylation Comparison Tool [19] was used to assess the correlations between methylation in blood and prefrontal cortex (PFC), entorhinal cortex (EC), superior temporal gyrus (STG), and cerebellum.

#### Postmortem brain DNAm

DNAm measured from post-mortem brains was obtained from two studies, each of which examined a unique but not necessarily distinct set of brain regions and cohorts: the National Center for PTSD Brain Bank cohort (NCPTSD-BB [88]) and the PsychENCODE Consortium for PTSD (PEC-PTSD) Brainomics cohort [89] (see Additional file 1: eMethods for details), both of which were sourced from the Lieber Institute for Brain Development.

Methylation at PTSD-associated CpGs identified in the EWAS was tested for association with PTSD in DNA extracted from postmortem dorsolateral prefrontal cortex (dlPFC, BA9/46), ventromedial prefrontal cortex (vmPFC, BA12/32), amygdala, and dentate gyrus (DG). DNAm in the post-mortem tissue was measured using the EPIC array. We examined the associations with PTSD in dlPFC and vmPFC of 42 PTSD cases and 30 controls from the NCPTSD-BB. The associations between DNAm and PTSD in amygdala and DG were tested in 77 PTSD cases and 77 controls from the PEC-PTSD. Hypergeometric tests were used to examine if the number of CpGs nominally associated with PTSD in both blood and brain tissues is more than expected by chance (see Additional file 1: eMethods for details).

#### Neuronal nuclei

We examined cross-tissue association from neuronal nuclei isolation from the orbitofrontal cortex (OFC) of 25 PTSD cases and 13 healthy controls collected at the VA’s NCPTSD-BB [90]. Fluorescence-Activated Nuclei Sorting (FANS) protocol was employed to isolate NeuN + cells and the nuclei underwent reduced representation oxidative bisulfite-sequencing (RRoxBS), as previously described [91] (see Additional file 1: eMethods for details). We examined whether there was differential methylation within 500 bp of CpGs from the epigenome-wide association analyses. Four CpG sites match between the EPIC array and RRoxBS within 500 bp (cg05575921, cg21161138, cg23576855, and cg26599989).

#### Cellular model of prolonged stress

Methylation at PTSD-associated CpGs identified in the EWAS was tested for association with prolonged stress in DNA extracted from fibroblasts obtained from the Coriell Institute Cell Repository. EPIC array was used to measure DNAm from a cellular model of prolonged stress in which fibroblasts were subjected to physiological stress hormone (cortisol) levels for a prolonged period (51 days) as previously described [92, 93]. Student’s *t*-test examined DNAm differences at PTSD-associated CpGs between cortisol (cellular model of prolonged stress) and vehicle (control) groups. Each treatment group included six biological replicates.

## Results

### Epigenome-wide association meta-analysis

**Fig. 1:**
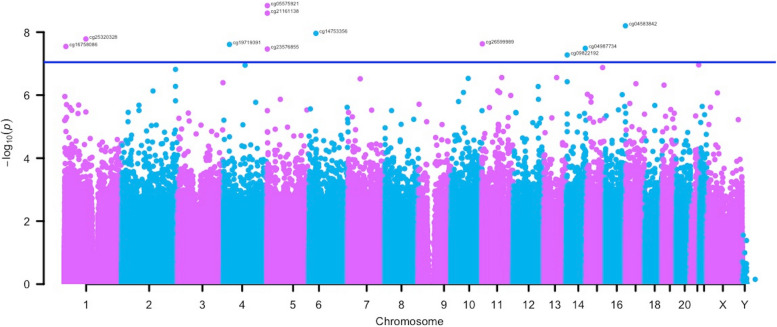
Manhattan plot of the epigenome-wide association meta-analyses. The *x*-axis depicts chromosomes and the location of each CpG site across the genome. The *y*-axis depicts the − log10 of the unadjusted *p*-value for the association with current PTSD. Each dot represents a CpG site. The solid blue line indicates the epigenome‐wide statistical significance at *p* < 9.0e − 8

We identified 11 PTSD-associated CpGs that passed the epigenome-wide significance threshold (5.44 <|*z*|< 6.05, 5.3e − 08 < *p* < 1.4e − 09), Table 2, Fig. 1, Additional file 1: Fig. S1). Two of the CpGs near *AHRR* (cg05575921 and cg21161138) were associated with PTSD in the previous meta-analysis from the PGC-PTSD Epigenetics Workgroup [31], while the other 9 were novel. All CpG sites, except one site (cg21161138) near *AHRR*, remained nominally significant (3.17 <|*z*|< 5.19, 1.52e − 03 < *p* < 2.10e − 07) with the same direction of association in the sensitivity analysis adjusted for smoking (Additional file 1: Table S1 and Fig. S1).

**Table 2:** CpG sites associated with current PTSD in the primary meta-analysis

CpG	Position	Gene	*β*	SE	*z*	*p*-value
cg16758086*	chr1: 6,173,356	*CHD5*	0.04	0.01	5.55	2.85e − 08
cg25320328	chr1:92,953,037	*GFI1*	− 0.03	0.01	− 5.65	1.64e − 08
cg19719391	chr4:26,789,915	*Intergenic*	0.03	0.01	5.58	2.45e − 08
cg23576855	chr5:373,299	*AHRR*	− 0.10	0.03	− 5.52	3.44e − 08
cg05575921	chr5:373,378	*AHRR*	− 0.04	0.01	− 6.05	1.44e − 09
cg21161138	chr5:399,360	*AHRR*	− 0.10	0.04	− 5.96	2.50Ee − 09
cg14753356	chr6:30,720,108	*Intergenic*	− 0.04	0.01	− 5.72	1.09e − 08
cg26599989	chr11:1,297,087	*TOLLIP*	0.03	0.01	5.58	2.35e − 08
cg04987734	chr14:103,415,873	*CDC42BPB*	0.03	0.01	5.53	3.26e − 08
cg09822192	chr14:24,801,191	*ADCY4*	0.04	0.01	5.44	5.30e − 08
cg04583842	chr16:88,103,117	*BANP*	0.04	0.01	5.81	6.29e − 09

Position is based on hg19. CpGs specific to EPIC-array were indicated with an asterisk (*). β, regression beta; SE, standard error

Of the 11 PTSD-associated CpGs, 9 CpGs were annotated to a gene expressed in blood. Meta-analysis across 4 cohorts with RNAseq data identified 5 CpGs whose methylation levels were correlated with their annotated gene expression (*p* < 0.05), but only 3 CpGs remained significant after multiple test corrections for 9 CpGs (*p*_Bonferroni_ = 0.05/9 = 5.5e − 03). Specifically, methylation of cg05575921, cg21161138, and cg23576855 associated with *AHRR* expression (*p* < 5.5e − 03, − 16.69 ≤ *z* ≤  − 4.86; Additional file 1: Table S2). These findings highlight the potential regulatory impact of these CpGs on gene expression in the context of PTSD. Evaluation of the top 1000 CpGs did not result in any significant Gene Ontology enrichments.

### Cross-tissue associations

**Fig. 2:**
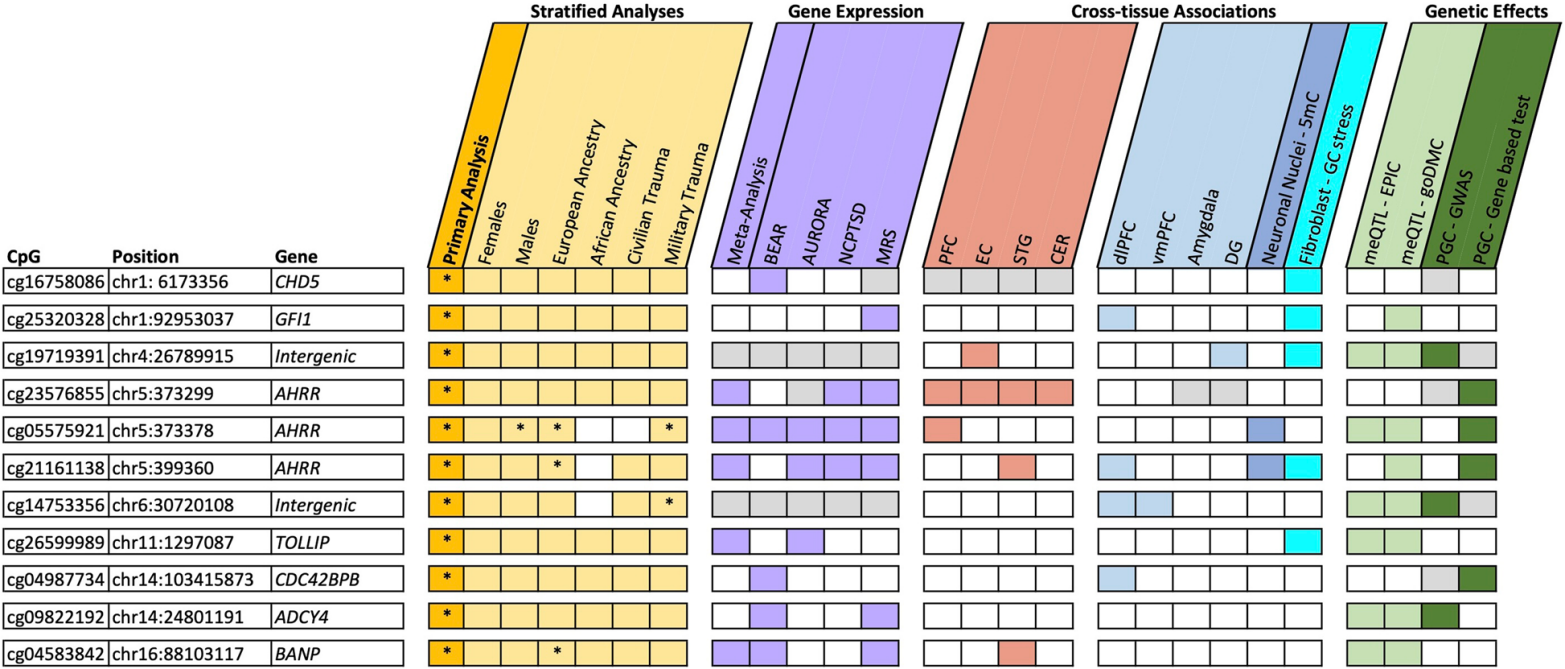
Summary of all analyses and findings. The figure combines CpGs from the main analysis (gold) and stratified analyses for sex, ancestry, and trauma type (light gold); and summarizes the results of blood and brain correlations (rose); gene expression (purple); cross-tissue associations for multiple brain regions (light blue), neuronal nuclei (blue), and a fibroblast model of prolonged stress (aqua); and genetic effects, including methylation quantitative trait loci (meQTL) analyses (light green) and genetic associations from the recent PGC-PTSD GWAS (dark green). Positive findings (*p* < 0.05) are indicated with the specific color of the respective category. Asterisk (*) indicates epigenome-wide significance (*p* < 9e − 8). Gray represents the CpGs or genes that were not present in the respective datasets. PFC, prefrontal cortex; EC, entorhinal cortex; STG, superior temporal gyrus; CER, cerebellum; dlPFC, dorsolateral prefrontal cortex; vmPFC, ventromedial prefrontal cortex; DG, dentate gyrus; 5mC, 5-methylcytosine; GC, glucocorticoid

We next sought to evaluate whether these blood-based associations may reflect PTSD-associated differences in the brain. Of the 11 PTSD-associated CpGs, 5 appeared to demonstrate some degree of correlation in at least 1 brain region (Fig. 2, Additional file 1: Table S3) based on data from the Blood Brain DNA Methylation Comparison Tool database [19]. The strongest correlation was observed between the blood and PFC (*r* = 0.91) for cg23576855 (*AHRR*). Such correlation could result from either a parallel response to an environmental stimulus, such as stress, or underlying genetic variation. To investigate whether these correlations might be driven by a ubiquitous stress response, we leveraged data from a naturalistic model of stress [92]. Four CpGs exhibited significant methylation changes in fibroblasts when subjected to cortisol in a cellular model of prolonged stress after a Bonferroni correction for 11 CpGs examined (0.057 < ΔDNAm < 0.225*, p* < 4.5e − 03, Fig. 2, Additional file 1: Table S4). For example, methylation of cg16758086 in *CHD5* increased more than 10% in response to cortisol (ΔDNAm = 0.101,* p* = 7.11e − 04).

When examining the possibility of genetic-epigenetic effects, we noted that 8 CpGs were associated with at least one SNP within 1 MB according to GoDMC [86] and meQTL EPIC [94] databases (Additional file 1: Table S5). In total, we identified 26 lead meQTLs that were tested for association in the latest PGC-PTSD GWAS [11], of which 8 were nominally associated with PTSD (*p* < 0.05; Additional file 1: Table S5). For instance, lower methylation at the intergenic cg14753356 site is associated with PTSD (*z* =  − 5.72, *p* = 1.09e − 08, Table 2) and higher methylation at cg14753356 is associated with rs28986310 T allele (beta = 0.31, *p* < 5e − 324), which increases the risk of PTSD (*z* = 6.73, *p* = 1.68e − 11, Additional file 1: Table S5). These data collectively suggest that some CpGs are under environmental influence, some are under genetic influence, and some are influenced by both genes and the environment. It is interesting to note that methylation of some CpGs, such as cg04987734 in *CDC42BPB*, did not appear to change in response to stress or to associate with underlying genetic variation. However, *CDC42BPB* did associate with PTSD in the recent PGC-PTSD gene-based analysis (*z* = 3.20, *p* = 6.94e − 04, Additional file 1: Table S6), suggesting that there are other mechanisms or perhaps tissue-specific regulation, underlying its association with PTSD.

We also investigated the cell type-specific expression patterns of the 7 genes identified in our study across various blood and brain cell types, using the Blood and Brain Atlas [95, 96]. The genes for which we observed a correlation between CpG methylation and expression in blood (*AHRR*, *TOLLIP*, *BANP*), and where methylation patterns were correlated between blood and brain (*AHRR*, *BANP*), were expressed across various immune cell types and brain regions. *CHD5*, *GFI1*, *ADCY4*, and *CDC42BPB* are expressed, albeit at low levels, across different immune cell types, and at high levels across all brain regions. In our study, the methylation of CpGs in these genes did not correlate with their expression in blood. Additionally, the methylation patterns of CpGs in these genes differed between blood and brain, potentially because these genes are not expressed in many blood cells.

### Associations in postmortem brain tissues

To evaluate whether the PTSD-associated CpGs from the blood-based analysis were also associated with PTSD in the brain regions of dlPFC, vmPFC, amygdala, and DG, we first examined overall patterns of association (*p* < 0.05) between blood-based PTSD-associated CpGs and those associated with PTSD in each of the respective brain regions using hypergeometric tests in probes with > 10% variability across tissues. The number of nominally significant CpGs associated with PTSD in both blood and the amygdala (*p* = 0.009) is more than expected by chance (Additional file 1: Table S7).

Next, we evaluated the specific PTSD-associated CpGs from blood-based analyses. Six CpGs were nominally associated with PTSD in at least one brain region (*p* < 0.05, Fig. 2, Additional file 1: Table S8). Notably, many of the CpG sites that changed in response to that naturalistic stress model associated with PTSD in at least one postmortem brain region (i.e., cg21161138 in *AHRR*, cg25320328 in *GFI*, and intergenic cg19719391). However, only cg04987734 (*CDC42BPB*) in the dlPFC and intergenic cg19719391 in the DG remained associated after applying a Bonferroni correction for the 11 CpGs examined (*p* < 4.5e − 3, Fig. 2, Additional file 1: Table S8). Specifically, PTSD cases had higher cg04987734 (*CDC42BPB*) methylation both in the blood (*p* = 3.26e − 8) and the dlPFC (*p* = 3.9e − 3), and higher and intergenic cg19719391 methylation both in the blood (*p* = 2.45e − 8) and the DG (*p* = 3.04e − 3) compared to trauma-exposed controls, suggesting a robust epigenetic alteration associated with PTSD that is detectable across different tissue types.

Finally, to more specifically examine the location of PTSD-associated CpGs in the brain, we leveraged data from FACS-sorted neuronal nuclei from the OFC of PTSD cases and controls. Of the 5 CpGs that associated with PTSD in any brain region from the bulk tissue, only CpGs in *AHRR* (cg05575921, cg21161138) appeared to differ in those with PTSD versus controls (Additional file 1: Table S9), suggesting that the other brain-based associations may be driven by other cell types, such as glia.

### Stratified analyses for sex, ancestry, and cohort type

To identify DNAm differences specific to a sex, genetic ancestry, or cohort type, stratified analyses were performed (Table 3, Additional file 1: Fig. S2). We examined the direction of effects of 4937 CpG sites that nominally associated (*p* < 0.05) with PTSD in both the European and African ancestry strata (Additional file 1: Fig. S3A) and 4265 CpG sites that nominally associated (*p* < 0.05) with PTSD in both the male and female strata (Additional file 1: Fig. S3B). While there were significant correlations in effect sizes across analyses stratified by ancestry (*r* = 0.48, *p* < 2.2e − 16) and sex (*r* = 0.32, *p* < 2.2e − 16), and directions of associations of the 11 PTSD-associated CpGs from the primary analysis were largely consistent across strata, some unique associations emerged (Additional file 1: Table S10). We identified 1 epigenome-wide significant CpG site (cg25691167 in *FERD3L*) associated with PTSD in the female-stratified analysis (*z* = 5.48, *p* = 4.24e − 8; Additional file 1: Fig. S4). The 1 significant CpG site (cg05575921 in *AHRR*) in the male-stratified analysis (*z* =  − 6.12, *p* = 9.3e − 10; Additional file 1: Fig. S5) was also identified in the primary analysis (Fig. 2). Five CpGs were associated with PTSD in European ancestry-stratified analysis (5.43 <|*z*|< 6.85, 5.7e − 08 < *p* < 7.2e − 12; Additional file 1: Fig. S6), of which 2 were unique and 3 were identified in the primary analysis (Fig. 2). One CpG (cg02003183 in *CDC42BPB*) was associated with PTSD in the African ancestry-stratified analysis (*z* = 5.48, *p* = 4.26e − 8; Additional file 1: Fig. S7). When examining cohort type, 1 CpG (cg27541344 in *BCL11B*) was associated with PTSD in the analysis of civilian cohorts (*z* = 5.39, *p* = 7.21e − 8; Additional file 1: Fig. S8). Finally, 5 CpGs were associated with PTSD in the military cohorts (5.36 <|*z*|< 6.22, 8.5e − 08 < *p* < 4.9e − 10; Additional file 1: Fig. S9), of which 3 were unique and 2 were identified in the primary analysis (Fig. 2).

**Table 3:** CpG sites associated with current PTSD in the stratified analyses

**CpG**	**Position**	**Gene**	**β**	**SE**	**z**	***p*-value**
*Stratified analysis for females*						
cg25691167	chr7:19184961	*FERD3L*	0.09	0.03	5.48	4.24E-08
*Stratified analysis for males*						
cg05575921	chr5:373378	*AHRR*	-0.14	0.04	-6.12	**9.30E-10**
*Stratified analysis for European ancestry*						
cg05575921	chr5:373378	*AHRR*	0.05	0.02	-6.85	**7.24E-12**
cg21161138	chr5:399360	*AHRR*	-0.16	0.05	-5.62	**1.95E-08**
cg11256214	chr12:110211642	*MGC14436*	0.06	0.02	5.76	8.17E-09
cg15977432	chr19:56709655	*Intergenic*	-0.05	0.02	-5.46	4.68E-08
cg04583842	chr16:88103117	*BANP*	0.05	0.02	5.43	**5.66E-08**
*Stratified analysis for African ancestry*						
cg02003183	chr14:103415882	*CDC42BPB*	0.11	0.04	5.48	4.26E-08
*Stratified analysis for civilian trauma*						
cg27541344*	chr14:99650422	*BCL11B*	0.02	0.04	5.39	7.21E-08
*Stratified analysis for military trauma*						
cg03329539	chr2:233283329	*Intergenic*	-0.03	0.01	-5.36	8.53E-08
cg21566642	chr2:233284661	*Intergenic*	-0.14	0.04	-5.41	6.36E-08
cg14753356	chr6:30720108	*Intergenic*	-0.04	0.02	-5.47	**4.53E-08**
cg05575921	chr5:373378	*AHRR*	-0.07	0.02	-6.22	**4.92E-10**
cg00774777*	chr11:76478902	*RP11-21L23.4*	0.03	0.01	5.55	2.79E-08

Position is based on hg19. The sites that were also epigenome-wide significant (*p* < 9e-08) in the primary meta-analysis were shown in bold. CpGs specific to EPIC-array were indicated with an asterisk (*). β: Regression beta. SE: Standard error.

We identified 3 GO term enrichments in the female-stratified analysis (FDR < 0.05), including nervous system development (Additional file 1: Table S11). We did not identify any significant enrichments in other strata. These findings suggest that PTSD-associated DNAm patterns related to nervous system development may exhibit sex-specific patterns. The absence of significant GO term enrichments in the main analysis or other stratified analyses highlights the potential importance of considering sex-specific factors in epigenomic studies.

## Discussion

In the present epigenome-wide meta-analysis of blood DNAm levels, we identified 11 CpG sites associated with PTSD, of which 2 had been identified in a prior meta-analysis of PTSD and 9 were novel. Because this study was conducted in blood, and PTSD is a brain-based disorder, we were interested in the degree to which we would observe the association of these blood-based CpGs in different brain regions implicated in PTSD along with other large-scale discoveries of PTSD. We noted overall enrichment of PTSD-associated CpGs in the amygdala and dentate gyrus. In addition, many loci showed blood–brain methylation correlations and cross-tissue associations with PTSD, with significant correlations between CpG site methylation levels and their respective gene expression levels.

We observed 3 CpGs (cg05575921, cg21161138, and cg23576855) in *AHRR* (aryl-hydrocarbon receptor repressor), 2 of which were identified in an earlier PGC-PTSD EWAS [31] and an independent study of US veterans [88]. DNA methylation at the *AHRR* CpGs is known to be influenced by smoking [97], and our effect sizes were attenuated when controlling for a DNAm-based smoking score. However, our previous study demonstrated that these associations were driven by non-smokers and were likely to be independent of smoking [31]. This is consistent with our finding that methylation of *AHRR* CpG sites changes in an in vitro model of naturalistic stress [92] and with the observation that *AHRR* associates with gene-based tests of PTSD from the PGC-PTSD GWAS [11]. The aryl hydrocarbon receptor (AhR) plays a role in immunomodulation, including the regulation of T lymphocytes, B cell maturation, and the activity of macrophages, dendritic cells, and neutrophils [98], supporting the link between the immune system and PTSD. Interestingly, *AHRR* methylation patterns in the blood of those with PTSD were associated with tryptophan metabolites, including the lower kynurenine and kynurenic acid levels [31]. Notably, cg21161138 DNAm was also associated with PTSD in postmortem dlPFC and neuronal nuclei from the OFC. Collectively, these data support that *AHRR* plays a role in PTSD that is independent of smoking status and warrants further mechanistic studies.

Our *CDC42BPB* (*CDC42 binding protein kinase beta*) findings are of particular interest. *CDC42BPB* is involved in the regulation of cytoskeletal rearrangement, cell migration, and neurodevelopment [99]. In the current study, increased *CDC42BPB* methylation at cg04987734 was associated with PTSD in both blood and the dlPFC. Notably, higher methylation at cg04987734 has been associated with depressive symptoms [100] and increased C-reactive protein (CRP) levels [101, 102], which is perhaps not surprising given the genetic correlation between PTSD and MDD [11] and the bi-directional genetic association between PTSD and CRP levels [103]. Multiple studies reported increased CRP levels and other inflammatory markers in those with PTSD, suggesting inflammation as an important component of PTSD [104–106]. Future studies are warranted to investigate the role of *CDC42BPB* in psychiatric disorders and the degree to which *CDC42BPB* methylation varies with respect to PTSD onset and treatment response.

We also identified PTSD-associated CpGs in genes *GFI1*, *CHD5*, *TOLLIP*, *ADCY4*, and *BANP*. While the precise mechanisms linking these genes to PTSD are not entirely understood, evidence suggests that they are responsive to stress and have been implicated in stress-related disorders, immune response, and other psychiatric disorders [107–111]. Collectively, these genes may contribute to PTSD through alterations in gene expression, synaptic and neural plasticity, and neuroimmune interactions. Future research should focus on elucidating the specific pathways and mechanisms by which these genes influence stress response and PTSD.

The stratified analyses identified DNAm-PTSD associations specific to sex, ancestry, and cohort type to identify DNAm differences that may be specific to strata, such as hormonal factors that underlie sex differences or occupational exposures related to military service. The PTSD-associated site cg25691167 (*FERD3L*) in females (*p* = 4.24e − 08) was not associated with PTSD in males (*p* = 0.57), suggesting that DNAm changes in cg25691167 might be sex-specific. The *FERD3L* (Fer3-like bHLH transcription factor) gene is a transcription factor involved in various developmental processes, particularly in neurogenesis [112], which is consistent with our pathway enrichment findings in the female-stratified analysis that identified nervous system development. Similarly, cg27541344 (*BCL11B*) was associated with PTSD in the civilian (*p* = 7.21e − 08), but not the military cohorts (*p* = 0.38), whereas 3 out of 5 PTSD-associated CpGs in the military cohorts were not significant in the civilian cohorts (*p* > 0.05). Though these associations are promising and warrant further study, it is important to note that the sex-stratified analyses may be confounded by the fact that many male-dominant cohorts are military cohorts, where specific factors such as circadian rhythm, age, and diet during military practices may drive the observed sex-specific findings.

### Strengths and limitations

To our knowledge, this is the largest EWAS of PTSD to date. Our sample is diverse in terms of sex, ancestry, and cohort type. We leveraged data from postmortem brain samples, a cellular model of prolonged stress, GWAS, and genome-wide expression data to support our findings. However, the study is not without limitations. First, methylation arrays only assess a subset of CpG sites in the genome; therefore, we may not capture all PTSD-associated CpG sites. Second, this is a cross-sectional study of participants with prior exposure to a traumatic event; thus, we were not able to assess whether the differences in DNAm between individuals with and without PTSD are a cause or consequence of PTSD or both. Third, our primary meta-analysis was performed using measures of blood DNAm. While this strategy provided valuable insights for future research on biomarkers of PTSD, it might not accurately represent the DNAm patterns within other tissues that are likely the most relevant to PTSD. However, the majority of the PTSD-associated CpG sites’ methylation levels were correlated between blood and at least one brain region. In addition, most PTSD-associated CpGs in blood were also associated with PTSD in one or more brain regions. Fourth, we do not have cell-type-specific DNAm and gene expression information necessary for further examination of cell-specific gene regulation across blood and brain cell types. Hence, we used bulk tissue and adjusted for cellular heterogeneity, which might have obscured some signals, given the alterations in cell composition in those with PTSD [113, 114]. Additionally, the brain regions examined varied between the online databases used to examine the blood–brain correlation of methylation values and the gene expression differences associated with PTSD, which can complicate interpretation. Finally, most cohorts that participated in the meta-analysis did not have detailed physical or psychiatric information on participants, including detailed information on chronicity, trauma type and timing, PTSD symptom course, and treatment, making it challenging to evaluate and adjust for potential confounders, including substance use, comorbidities, or medication use. The lack of detailed information on the types and timing of traumatic experiences across cohorts prevents us from making definitive conclusions about the impact of different trauma types on DNAm. Future studies are warranted to examine DNAm changes longitudinally, tracking participants both before and after trauma exposure to capture the dynamic epigenetic modifications that occur in response to traumatic events, providing a clearer understanding of how trauma influences DNAm over time.

## Conclusions

Taken together, this study replicates our previous findings and identifies novel PTSD-associated CpGs. Supporting data from multiple sources suggest that epigenetic mechanisms, particularly methylation in *AHRR* and *CDC42BPB*, may contribute to the complex relationship between the immune system and PTSD.

## Supplementary Information

Additional file 1: Table S1. PTSD-associated CpGs from the primary analysis in the sensitivity analysis adjusted for smoking score. Table S2. DNA methylation and cis-gene expression (RNAseq) correlations. Table S3. Correlation values of methylation between blood and brain regions from Blood Brain DNA Methylation Comparison Tool. Table S4. Results of PTSD-associated CpGs from blood in fibroblasts. Table S5. Significant SNPs from cis-meQTL analysis and their association with PTSD. Table S6. Genetic associations of the identified PTSD-associated genes from gene-based tests. Table S7. Results of hypergeometric tests in postmortem brain tissues. Table S8. Results of PTSD-associated CpGs from blood in the brain. Table S9. Results of PTSD-associated CpGs from blood in neuronal nuclei. Table S10. PTSD-associated CpGs from the primary analysis in the stratified analysis for sex, ancestry, and trauma type. Table S11. Gene Ontology Enrichment results in females. Fig S1. Forest Plots of PTSD-associated CpGs in each cohort. Fig S2. Manhattan plots of the stratified analyses. Fig S3. Correlation of effect sizes based on stratified analyses for ancestry and sex. Fig S4. Forest Plots of CpGs associated with PTSD in the stratified analysis for females. Fig S5. Forest Plots of CpGs associated with PTSD in the stratified analysis for males. Fig S6. Forest Plots of CpGs associated with PTSD in the stratified analysis for European ancestry. Fig S7. Forest Plots of CpGs associated with PTSD in the stratified analysis for African ancestry. Fig S8. Forest Plots of CpGs associated with PTSD in the stratified analysis for civilian trauma. Fig S9. Forest Plots of CpGs associated with PTSD in the stratified analysis for military trauma.

Additional file 2. Summary statistics of the main analysis.

Additional file 3. Summary statistics of the smoking-sensitivity analysis.

Additional file 4. Summary statistics of the stratified analysis for males.

Additional file 5. Summary statistics of the stratified analysis for females.

Additional file 6. Summary statistics of the stratified analysis for European ancestry.

Additional file 7. Summary statistics of the stratified analysis for African ancestry.

Additional file 8. Summary statistics of the stratified analysis for civilian cohorts.

Additional file 9. Summary statistics of the stratified analysis for military cohorts.

## Data Availability

The main summary statistics data that support the findings of this study will be available within Supplementary Data upon publication. Individual-level data from the cohorts or cohort-level summary statistics will be made available to researchers following an approved analysis proposal through the PGC-PTSD Epigenetics Workgroup with the agreement of the cohort PIs. The raw data for the GTP cohort is available in the Gene Expression Omnibus database with the accession code GSE132203 [115]. Owing to limitations on data sharing as specified in the consent process and/or restrictions as part of the use agreement under which the data was accessed, data from the military/VA cohorts, VA MIRECC, MRS, Army STARRS, PRISMO, PROGrESS, and NCPTSD/TRACTS, cannot be publicly posted as part of this project. However, such data can be provided in de-identified form through a data use agreement following applicable guidelines on data sharing and privacy protection. For additional information on access to these data, including PI contact information for the cohorts accessed under a DUA, please contact the corresponding author.

## Abbreviations

CpG: Cytosine-guanine dinucleotides
CRP: C-reactive protein
DG: Dentate gyrus
dlPFC: Dorsolateral prefrontal cortex
DNAm: DNA methylation
EC: Entorhinal cortex
EWAS: Epigenome-wide association study
FANS: Fluorescence-Activated Nuclei Sorting
FDR: False discovery rate
GWAS: Genome-wide association study
IVW: Inverse-variance weighted
meQTL: Methylation quantitative trait locus
NK: Natural killer
OFC: Orbitofrontal cortex
PC: Principal component
PFC: Prefrontal cortex
PGC-PTSD: Psychiatric Genomics Consortium PTSD Workgroup
PTSD: Posttraumatic stress disorder
QC: Quality control
RPC: Robust partial correlation
RRoxBS: Reduced representation oxidative bisulfite-sequencing
STG: Superior temporal gyrus
vmPFC: Ventromedial prefrontal cortex
